# Combining crystallographic and binding affinity data towards a novel dataset of small molecule overlays

**DOI:** 10.1007/s10822-024-00581-1

**Published:** 2024-12-04

**Authors:** Sophia M. N. Hönig, Torben Gutermuth, Christiane Ehrt, Christian Lemmen, Matthias Rarey

**Affiliations:** 1 BioSolveIT, An der Ziegelei 79, 53757 Sankt Augustin, Germany; 2 University of Hamburg, ZBH - Center for Bioinformatics, Albert-Einstein-Ring 8-10, 22761 Hamburg, Germany

## Abstract

**Abstract:**

Although small molecule superposition is a standard technique in drug discovery, a rigorous performance assessment of the corresponding methods is currently challenging. Datasets in this field are sparse, small, tailored to specific applications, unavailable, or outdated. The newly developed LOBSTER set described herein offers a publicly available and method-independent dataset for benchmarking and method optimization. LOBSTER stands for “Ligand Overlays from Binding SiTe Ensemble Representatives”. All ligands were derived from the PDB in a fully automated workflow, including a ligand efficiency filter. So-called ligand ensembles were assembled by aligning identical binding sites. Thus, the ligands within the ensembles are superimposed according to their experimentally determined binding orientation and conformation. Overall, 671 representative ligand ensembles comprise 3583 ligands from 3521 proteins. Altogether, 72,734 ligand pairs based on the ensembles were grouped into ten distinct subsets based on their volume overlap, for the benefit of introducing different degrees of difficulty for evaluating superposition methods. Statistics on the physicochemical properties of the compounds indicate that the dataset represents drug-like compounds. Consensus Diversity Plots show predominantly high Bemis–Murcko scaffold diversity and low median MACCS fingerprint similarity for each ensemble. An analysis of the underlying protein classes further demonstrates the heterogeneity within our dataset. The LOBSTER set offers a variety of applications like benchmarking multiple as well as pairwise alignments, generating training and test sets, for example based on time splits, or empirical software performance evaluation studies. The LOBSTER set is publicly available at https://doi.org/10.5281/zenodo.12658320, representing a stable and versioned data resource. The Python scripts are available at https://github.com/rareylab/LOBSTER, open-source, and allow for updating or recreating superposition sets with different data sources.

**Graphical abstract:**

Simplified illustration of the LOBSTER dataset generation.
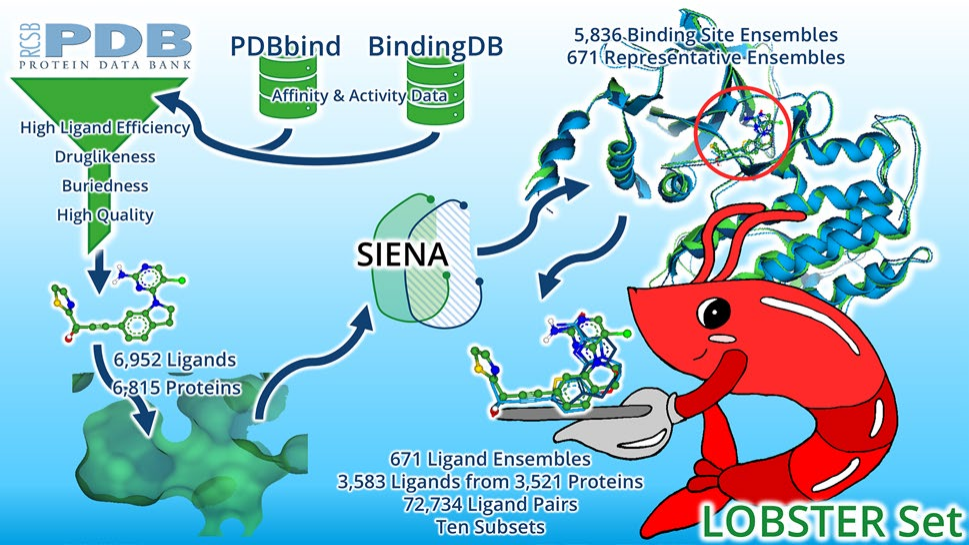

**Supplementary Information:**

The online version contains supplementary material available at 10.1007/s10822-024-00581-1.

## Introduction

Drug discovery and design methods rely heavily on appropriate benchmark data for development, rigorous assessment, and comparison. However, many datasets are sparse and/or tailored to the specifics of a particular method. Therefore, a fair and unbiased comparison of new approaches with existing methods is difficult. Small molecule superposition is a standard technique in drug discovery as it is a prerequisite for the visual comparison of molecules and many applications like modeling 3D Quantitative Structure-Activity Relationships (QSAR) or pharmacophore elucidation. Aligning two or more molecules in a coordinate space based on their atomic coordinates, volume, or other three-dimensional descriptors leads to a superposition. To the best of our knowledge, no suitable dataset is currently available and consistently used to benchmark novel superposition methods, although it is of fundamental importance. [1] One reason may be that it is difficult to define a ground truth due to many different scoring schemes. For example, superpositions based on a common substructure might be fundamentally different from superpositions based on the volume or the pharmacophoric properties of the solvent-exposed substituents of a common scaffold. Table 1 provides a comprehensive overview of superposition performance-related publications and the size of the underlying dataset, the considered tools, and the data availability.

**Table 1:** Selected list of benchmark publications comparing multiple tools for small molecule superposition

Reference	Year	Molecules	Tools	Available
**Chen et al.** [2]	2006	145	FlexS [3–5], ROCS [6, 7]	X
Hawkins et al. [8]	2007	1000	FlexS [3–5], ROCS [6, 7]	X
**Marialke et al.** [9]	2007	19	GMA [9], FlexS [3–5], fFlash [10]	X
Giganti et al. [11]	2010	1100	Surflex-Sim [12, 13], FlexS [3–5], ROCS [6, 7], ICMsim (APF) [14, 15]	$\checkmark$ [16, 17]
Venkatraman et al. [18]	2010	> 100,000	ESHAPE3D (MOE) [19, 20], ROCS [6, 7], PARAFIT [21], ShaEP [22], USR [23]	$\checkmark$ [16, 17]
**Giangreco et al.** [24]	2013	1464	SENSAAS [25], SENSAAS-Flex [26]	$\checkmark$ [27]
**Kawabata and Nakamura** [28]	2014	636	FKCOMBU [28, 29], ShaEP [22]	$\checkmark$ [30]

Although superposition is also used in pharmacophore identification, we focus solely on dedicated small molecule superposition tools. Available datasets are marked and the corresponding reference to a download page is provided. Some benchmarks are based on screening datasets. Therefore, we highlighted the benchmarks assessing the geometric accuracy of molecular superpositions in bold

To assess the geometric accuracy of rigid and flexible 3D molecular superpositions by evaluating FlexS [3–5] and ROCS [6, 7], Chen et al. [2] created a dataset based on the crystal structures of 145 compounds from eight different proteins. For four of them, 111 compounds were superimposed by aligning the protein structures. The other 34 ligand alignments were derived from the manually curated FlexS-77 dataset by Lemmen et al. [3] The FlexS-77 set consists of 984 ligands intentionally chosen to have a limited number of low-energy conformations. One year later, Hawkins et al. [8] re-evaluated FlexS and ROCS to compare shape-matching and docking applications. Focusing on GPCR targets, 50 active and 950 decoy compounds were derived from MDDR. [31] A conformer database was generated and the superposition tools’ virtual screening performance was assessed. However, the tools have not been tested with regard to the quality of the generated superpositions, as there were no crystal structures to determine the actual binding conformation of the ligand. In the same year, Marialke et al. [9] used the dataset originally published with fFlash [10] to evaluate superpositions generated by GMA [9], FlexS, and fFlash. This dataset is a subset of the FlexS-77 dataset. The fFlash set consists of 19 compounds, each known to bind one of seven different proteins, each provided with coordinates from the crystal structure of the protein-ligand complex. While all three above-described studies offer a good starting point for further comparisons, all associated datasets became unavailable over time.

The Directory of Useful Decoys (DUD) [16, 17] offers a large, curated, and publicly accessible resource for virtual screening performance testing. DUD comprises known active molecules for serveral targets and similar molecules of unknown activity as decoys. Based on enrichment factors in a virtual screening campaign on the DUD dataset, Venkatraman et al. [18] evaluated several methods, including five superposition tools. Besides the crystal pose of each query ligand, a database with 1000 conformers per ligand was utilized. DUD is not suitable for benchmarking the geometric accuracy of small molecule alignments. The DUD-E [32] dataset offers an improved and rebuilt version of the DUD dataset that addressed shortcomings. The Demanding Evaluation Kits for Objective In silico Screening (DEKOIS) [33] is another approach for building decoy sets tailored to a given set of bioactive molecules. This approach facilitates generating new compound collections and enables the enhancement of existing collections with complementing data.

Almost simultaneously, Giganti et al. [11] evaluated superposition and docking tools on the active compounds of eleven proteins from DUD. A visual score was introduced to assess superposition accuracy by assigning the results to one of three categories: accurate superposition of most pharmacophoric points, orientation similar to the reference and at least 50 % of pharmacophoric points superimposed, or none of the above. The score describes the percentage of the active compounds assigned to each category.

The PharmBench set was created by Cross et al. [34] to offer a dataset similar to DUD for pharmacophore elucidation. For the refined PharmBench set, protein-ligand complexes from DrugPort [35] were selected by a well-described filtering protocol. A set of DNA major groove binders and five targets from a prior benchmark of pharmacophore identification tools by Patel et al. [36] were added in a final step. The PharmBench set comprises 81 targets and 960 ligands. Since pharmacophore elucidation also relies on superposition quality, ligand coordinates were derived from the aligned crystal structures of the target proteins to offer a basis for objective metrics to evaluate experiments. However, to the best of our knowledge, PharmBench was not used to benchmark superposition tools and is no longer available.

In a parallel development towards the same goal, the AstraZeneca (AZ) dataset was created by Giangreco et al. [24] Given the RCSB Protein Data Bank (PDB) [37, 38] as a starting point, several filters were applied to the crystal structures of the protein-ligand complexes in a highly automated selection to achieve the least possible subjectivity in ligand selection. To generate ligand overlay sets suitable for evaluating superposition tools, the respective protein structures were superimposed by their PROSITE motif [39]. Based on the assumption that large datasets are too challenging for pharmacophore elucidation, the overlay sets were reduced by retaining at most 40 ligands with the most interactions with protein residues. The resulting 121 publicly available ligand overlay sets, comprising 1464 compounds, were utilized for benchmarking SENSAAS [25] and SENSAAS-Flex [26].

One year later, Kawabata and Nakamura [28] created four datasets based on the PDB to compare their method FKCOMBU to the tool ShaEP. [22] Four proteins were chosen and protein-ligand complex structures with a sequence identity up to 95 % were assigned to the corresponding protein. The assigned proteins were superimposed and, in an attempt to provide overlays, refined by a filtering protocol for the included compounds. A superposition was considered successful if a generated conformer for a given reference structure could reproduce the crystal structure pose from the overlay by less than 2 Å.

The advances in the design of benchmark datasets, especially the publications of PharmBench and the AZ dataset, show a clear trend towards data refinement by (automated) filtering protocols and the impact of public availability on the usage of a dataset. Driving forces in the development of these benchmarks appear to be the objectivity of the metrics utilized for evaluations as well as the size and composition of the datasets. Some tools were commonly used in several benchmark experiments, e.g., FlexS and ROCS were analyzed in four benchmarks mentioned above. However, their performance was evaluated with different metrics, i.e., the RMSD to the crystal structures [2, 9], the area under the ROC curve [8], enrichment factors [18], or a score based on visual inspection [11]. Thus, the degree of difficulty of the respective dataset is hard to compare between the different benchmark approaches.

Following up on the key findings of the recent past, we present the LOBSTER dataset (“Ligand Overlays from Binding SiTe Ensemble Representatives”) of ligand overlays to evaluate small molecule superposition tools. Starting with all structures from the PDB available on November 7, 2023, the dataset generation and filtering protocols are fully automated to avoid subjectivity in the process of selecting protein-ligand complexes and to gain the largest possible set of compounds. All filter criteria were derived from the literature as outlined below. Affinity and activity data have been considered by selecting ligands with high ligand efficiency. Ligands were superimposed in their crystal orientation and conformation by aligning the corresponding binding pockets, resulting in structure ensembles. For poses generated in benchmark experiments, this offers an objective comparison to the superimposed ligand crystal poses. A clustering of ensembles created with the same protein-ligand complexes avoids redundancy within the LOBSTER set. The 671 ligand ensembles comprise 3212 unique ligands from 3521 protein-ligand complexes.

In this study, we propose a high-quality, diverse, and easily applicable dataset with the possibility to apply objective evaluation metrics. We hope this set will facilitate further benchmark experiments and studies and assist developers in rigorously evaluating their novel small molecule superposition methods.

## Methods

In short, the automated workflow for generating the LOBSTER dataset consists of four steps: First, all ligands are extracted from all PDB files of the RCSB Protein Data Bank (PDB) [37, 38] (Ligand extraction). Second, the ligands are filtered by ligand efficiency (LE) and molecular properties (Ligand selection). Then, ligands binding to identical pockets were superimposed by aligning their binding sites (Ensemble generation). A set of superimposed binding sites with an identical amino acid sequence is denoted as a binding site ensemble [40]. The resulting alignment of ligands is referred to as (ligand) ensemble. Finally, ensembles are grouped into clusters. Only one representative ensemble is picked from each cluster (Ensemble clustering). This fourth step prevents duplicate overlays resulting from redundant subpockets. We will comprehensively describe the four steps of the LOBSTER dataset generation below.

### Ligand extraction

The starting point of our workflow is an image of the PDB available on November 7, 2023. Protein structure information, including electron density support, is calculated by StructureProfiler [41]. For all considered ligands, molecular properties, like the number of rotatable bonds or the molecular weight, are calculated using the NAOMI framework [42, 43]. Among others, metal ions and covalent ligands were out of scope for this study and were therefore excluded from the calculations of the NAOMI framework (skipped by construction, $> 1.5$ Mio. sites). A detailed list of exclusion reasons is provided in the Supplementary Information (SI). A similar processing workflow was utilized for scanning the PDB structures to generate the PDBScan22 dataset [44]. The initial protein-ligand extraction process led to 753,050 binding sites for further consideration.

### Ligand selection

In the next step, drug-like small molecules with a high LE for at least one structure in the PDB are selected. Following the trend [45] to include compounds beyond the Rule of Five [46], we designed less strict filter criteria for drug-likeness to create the largest possible yet adequate dataset. These criteria were derived from multiple sources, as detailed below. An overview of the filtering steps and the number of remaining ligands is provided in Table 2.

**Table 2:** Filter criteria applied to the ligands and their respective structures

Step	Filter	Entries discarded	Entries remaining
0	–	–	2,436,944
1	No ligand	70,801	2,366,143
2	Skipped by construction	1,613,093	753,050
3	${\text {PDB Resolution}}> 2.5$ Å	146,616	606,434
4	$\textrm{EDIA}_{\textrm{m}} < 0.8$	433,150	173,284
5	Molecular weight $> 975\,\hbox {g mol}^{-1}$	372	172,912
6	Rotatable bonds $> 10$	14,502	158,410
7	Atom type filter	533	157,877
8	Heavy atoms $< 10$	99,030	58,847
9	Duplicate removal	23,383	35,464
10	No activity value	19,630	15,834
11	Ligand efficiency $\le 0.3$	8824	7010
12	Buriedness $< 0.5$	58	6952

An entry corresponds to one ligand of a PDB structure or the PDB structure itself if no ligands were extracted. Ligands that were out of scope for this study were labeled “Skipped by construction”. A detailed explanation for these cases is provided in the Supplementary Information. Only the instance with the best electron density score EDIA_m_ among all ligands with identical SMILES is kept during duplicate removal

The only global criterion for the protein structure quality was a resolution of at most 2.5 Å.

The EDIA score [47] quantifies the reliability of the ligand atoms’ fit into the experimental electron density. Only ligands with an $\textrm{EDIA}_\textrm{m}$ greater than 0.8 were retained, as recommended in the original publication.

Studies on the relationship between size and cell permeability specified an upper limit for molecular weight at $975\,\hbox {g mol}^{-1}$ for acceptable permeability [48]. To include this threshold as a filter criterion, molecules with a molecular weight of at most ${975}\,\hbox {g mol}^{-1}$ were accepted. A more permissive filter than in other datasets [24, 34] was chosen since the molecular weight of drugs is steadily increasing with the approval of active substances such as natural products and peptides [49, 50].

To exclude lipids and other highly flexible molecules, at most ten rotatable bonds were tolerated inside the molecule following Veber’s Rule for oral bioavailability [51]. Our definition of rotatable bonds matches the definition of Veber et al. (2002): A bond is considered rotatable if it is an acyclic single bond but no amide bond, no bond to a nitrile group, and no bond to a terminal heavy atom.

Molecules with elements other than C, O, N, S, P, Cl, F, Br, I, or B were excluded. Boron, which is not considered a standard organic atom [24], was included due to its increasing appearance in ligands addressing new targets and the tolerability of its toxicity [52].

To exclude most solvents, buffer molecules, and small ions, ligands with less than ten heavy atoms were removed from the dataset. This threshold was previously found to be effective for generating other datasets [24, 53].

In several cases, multiple instances of identical protein-ligand complexes exist. In these cases, only the ligand with the highest $\textrm{EDIA}_{\textrm{m}}$, i.e., the best fit to the electron density map, was kept for the respective PDB entry.

The pipeline to find complexes with high LE binders involves an automated parsing of the PDBbind [54, 55] and the BindingDB [56]. Since the PDBbind is a subset of the PDB, there is a direct mapping of the binding affinities or activities to the respective PDB entry and HET code of the ligand. The mapping in the BindingDB is established via sequence comparisons. According to the BindingDB website [57], for a mapping the ligands in both database entries must be identical, and the BindingDB target must have a sequence identity of at least 85 % to the protein chain in the PDB entry. However, to the best of our knowledge no further details for example on the used sequence (ATOM sequence, SEQRES sequence) or the molecular comparison (dissimilarities arising from differences in protonation) are provided. The BindingDB retrieves molecular activity and affinity data from various data sources. We want to emphasize that the choice of data sources might impact the accuracy and comprehensiveness of assay annotations [58, 59]. In particular, the provided metadata is often insufficient to, e.g., reliably exclude pathway-inhibition assays whose readout cannot be attributed to a compound’s activity on a single protein target or cell-based assays that often require tedious post-processing pipelines to exclude false positives (see SI for more details). However, since we are considering both structural and activity/affinity data simultaneously, we are confident that the conclusions on ligand binding are sufficiently robust.

Activity values of the type $\textrm{IC}_{50}$, $\textrm{EC}_{50}$, or $\textrm{K}_i$ and affinity values of the type $\textrm{K}_d$ are included. The values are normalized by $p({\textit{value}}) = -{\textit{log}}_{10}({\textit{value}})$. The term to calculate this value is derived from Bento et al. [60]. Please note that we avoid comparing the activity and affinity values from different sources. Instead, we use the values as a common ground to assess the minimum estimated LE of a ligand. Therefore, only the lowest value is used to calculate the LE. For a given value, the formula for calculating the LE [61] of a ligand *l* with a measured affinity or activity value (${\textit{value}}_l$) and heavy atom count (${\textit{hac}}_l$) is the following:

$${\textit{LE}}(l) = \frac{1.37 \cdot p({\textit{value}}_l)}{{\textit{hac}}_l}$$

Only ligands with a LE exceeding 0.3 are accepted according to the threshold provided by Cavalluzzi et al. [62].

In the last filtering step, the buriedness of each ligand within its protein binding site is calculated with an in-house application to exclude surface binders from the LOBSTER set. Applying the threshold formerly used to generate the sc-PDB [63], ligands that are less than 50% buried by protein atoms are discarded.

In total, the refined selection of compounds comprises 6952 ligands from 6815 protein structures.

### Ensemble generation and clustering

To automatically generate binding site ensembles from our filtered set of ligands, the tool SIENA [40] was utilized. SIENA was applied using the settings specified in the SI. For each ligand, the binding pocket was defined by all residues with at least one atom within 6.5 Å of any ligand atom. Each pocket served as a query for SIENA to search a database of protein structures that contain at least one ligand that passes our filter criteria. All binding sites with identical sequences are extracted. For each query, the resulting binding sites from the SIENA search were superimposed based on the binding site backbone atoms. Thus, the ligands were superimposed indirectly by the alignment of the binding sites. The aligned ligands were extracted as ligand ensembles. For each ensemble, we denote the ligand whose pocket was used for the SIENA search as a search ligand. Initially, 6935 ligand ensembles were generated. If multiple ligands with the same unique SMILES were assigned to the same ensemble, the ligand from the structure with the lowest binding site backbone RMSD was retained. Ensembles containing only one ligand after the deduplication were removed, reducing the number of ensembles to 5836.

Ensuring identical binding site sequences via SIENA, the aligned binding sites most likely also adopt a similar three-dimensional structure. There might be cases with diverging shapes. However, in our opinion, superposition tools should be capable of handling ligand conformations extracted from conformationally flexible binding sites. Thus, conformational flexibility was embraced within the ensemble generation by using the backbone RMSD.

Since differently sized and chemically diverse ligands can bind in partially overlapping sites of a binding pocket, selecting binding residues within 6.5 Å of each ligand will end up in multiple SIENA queries describing different parts of the pocket. To overcome this issue and keep the LOBSTER set as diverse as possible, we used a clustering procedure to retain only non-intersecting binding site ensembles (NBSE) [40]. In this context, non-intersecting means that the same protein-ligand complex should not be part of different ensembles. Otherwise, these ensembles would intersect and thereby describe the same binding pocket. Consequently, ensembles that share at least one protein-ligand complex are grouped into clusters. Algorithm 1 describes the clustering process as pseudocode.

**Algorithm 1:**
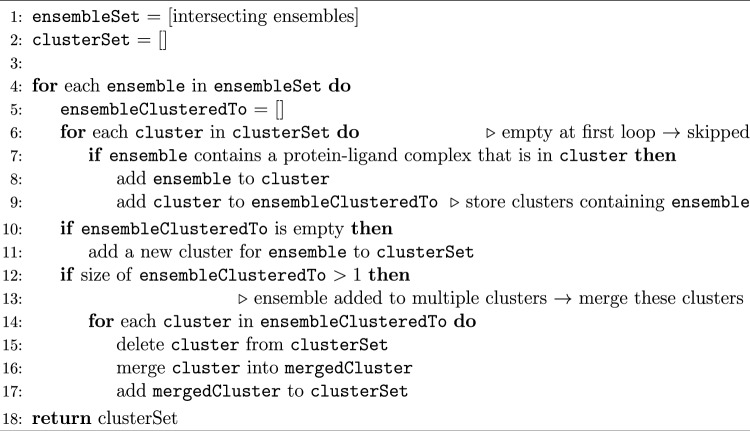
Algorithm for grouping intersecting binding site ensembles into clusters. Two ensembles are intersecting if they have a protein-ligand complex in common.

The algorithm depends on the order in which the ensembles are processed. This order affects the number of clusters in the outcome due to the merging procedure described in line 13 and following. By first processing the ensembles with the most ligands, we generate the largest possible number of clusters and, thereby, the largest set of non-intersecting ensembles. If ensembles have the same size, the ensemble name is considered for a tie-break.

Among all ensembles in one cluster, the ensemble resulting from the search ligand with the highest heavy atom count is chosen as a so-called cluster representative. Based on the assumption that larger binding sites may connect several subpockets, this approach ensures larger ensembles. The set of the 671 representatives describes the LOBSTER dataset.

### Comparison to approved drugs

To compare the distribution of molecular properties among the compounds in LOBSTER to those of orally available drugs, we used the list of known drugs with an oral route of administration from the FDA Orange Book with at least ten atoms [64]. For this purpose, the compounds from the Orange Book were assigned to structures of approved drugs from DrugBank [65] by name matching (correspondence of the names stored as “GENERIC_NAME”, “JCHEM_TRADITIONAL_IUPAC”, “SYNONYMS”, “INTERNATIONAL BRANDS”, or “PRODUCTS” from DrugBank to either the active ingredient or the proprietary name from the FDA Orange Book). The molecular weight, number of rotatable bonds, SlogP, and the number of hydrogen bond donors and acceptors were calculated with RDKit [66] in KNIME 5.3.0 [67].

### Subsets of ligand pairs

Since most superposition tools are limited to pairwise alignments [1], we combined ligands belonging to the same ensemble pairwise to extend the scope of the LOBSTER set. One ligand in a pair is considered the template ligand, while the other is called the query ligand. Thus, for two ligands *a* and *b* of the same ensemble, both pairs, template *a* with query *b* and template *b* with query *a* are considered. In an application scenario, the query ligand would be superimposed onto the template ligand. Since the ligand pairs were derived from binding pockets with identical sequences, a high similarity of the pockets is anticipated. Therefore, the ligands’ coordinates are assumed to deliver a reliable and biologically relevant superposition of the ligands. For each pair of molecules, the quality of the overlay is rated by the Shape Tversky Index calculated with RDKit version 2022.09.1 [66]. For a template ligand *T* and a query ligand *Q*, the index is calculated as follows:

$$\begin{aligned}&{\textit{ShapeTversky}}(T, Q)\\&\quad = \frac{{\textit{overlap}}(T, Q)}{{\textit{overlap}}(T, Q) + t \cdot {\textit{nonOverlap}}(T) + q \cdot {\textit{nonOverlap}}(Q)} \end{aligned}$$

The factors of this equation are chosen $t = 1$ and $q = 0$ since we want to evaluate how much of the template molecule is covered by the query molecule. We hypothesize that the closer the index is to one, i.e., the more the query shape overlaps with the template shape, the easier the query can be superimposed onto the template by scoring functions in a way that equals the biologically meaningful alignment. This measure provides an objective assessment of the difficulty of a superposition.

The dataset was split based on the pairwise Shape Tversky Indices with a step size of 0.1, i.e., the set *subset_80* contains all pairs with an index of $0.8 \le x < 0.9$. Of these subsets, *subset_90* is considered the easiest since the index of all pairs is at least 0.9.

### Calculation of consensus diversity

A Consensus Diversity Plot (CPD), as proposed by González-Medina et al. [68], shows the median MACCS fingerprint Tanimoto similarity on the x-axis in ratio to the Scaffold AUC on the y-axis for a given set of molecules. It thereby gives an impression of the diversity in terms of features as calculated through the fingerprint similarity while also showing the novelty of the compounds in terms of the Bemis–Murcko scaffolds. CPDs were generated for the LOBSTER dataset, the subsets generated from LOBSTER, and the AstraZeneca (AZ) dataset [24]. For this purpose, the MACCS fingerprint Tanimoto similarity and the Bemis–Murcko scaffolds were calculated using RDKit [66] version 2022.09.1. For the calculation of the AUC, we utilized scikit-learn [69] version 1.1.3.

### Extraction of UniProt and Pfam IDs from LOBSTER and AstraZeneca datasets and protein class assignment

Besides several statistics, a more sophisticated analysis of the protein diversity was conducted. Since we consider the AstraZeneca (AZ) dataset of Giangreco et al. [24] as a previous state-of-the-art dataset for overlays of ligands’ crystal poses, we compared the diversity of proteins from which the ligands were derived for the LOBSTER and the AZ dataset.

In the first step, the exact protein-ligand complexes used by both datasets were extracted. While this step was trivial for the LOBSTER dataset, only the ligands’ three-letter residue names and coordinates are supplied for the AZ set. For PDB entries with multiple ligands with the same residue name, we chose the reference ligand as follows. PyMOL [70] was used to extract all ligands. UNICON [71] was applied to interpret the ligands and add hydrogen atoms. Next, REMUS [72] was used to find the ligand with the lowest RMSD to the one provided in the AZ dataset. In several cases, REMUS could not be applied due to alternate conformations or differences in bond types. Therefore, these entries were manually investigated to find the best matching ligand. The data for this analysis can be found versioned with the LOBSTER dataset in Zenodo. To extract only relevant UniProt accession numbers, the interacting chains as calculated by the StructureProfiler [41] were used to query the PDB Data API [73] for UniProt accession number(s) corresponding to the identifiers of the interacting chain(s). Some PDB entries in the AZ dataset are already obsolete, so these PDB entries had to be substituted by their successors. In addition, the original PDB files were used for both datasets. Original files annotate the chain ID assigned by the author (“auth_asym_id”); the PDB Data API uses the PDB-assigned chain ID (“label_asym_id”). Any altered chains had to be changed accordingly to allow for automated analyses. The resulting data for both datasets can be found in Zenodo as well. For any UniProt accession number, the Pfam ID and the Pfam name were extracted using the Pfam Data API [74]. If no UniProt accession number is provided for an interacting chain, it is omitted from the analysis. In the LOBSTER dataset, the UniProt accession numbers for the entries in eight clusters are not annotated in the PDB. These clusters are omitted from the protein diversity analysis. Notably, all but two UniProt accession numbers annotated in the AZ dataset could be reconstructed using this automated analysis, with the two mismatched UniProt accession numbers no longer being supported (P0A5J2:P9WK19 and P0C5C1:P9WKD3). Protein classes were assigned manually based on Pfam and chain IDs, and a complete table is provided with the LOBSTER dataset. A detailed description of the assignment of protein classes based of Pfam IDs is available, too.

## Results

The LOBSTER dataset was designed for the alignment of multiple drug-like ligands. First, we compare the distributions of the molecular properties and flexibility from the LOBSTER compounds to orally bioavailable drugs. Next, the impact of the clustering to avoid overlapping protein pockets for non-intersecting ensembles is evaluated. Furthermore, the diversity of the LOBSTER set is analyzed for different degrees of difficulty and compared to the AstraZeneca (AZ) dataset [24]. The distribution of the data within the subsets is evaluated as well. Finally, the growth of the LOBSTER set over time is investigated by analyzing the date of first revision for the underlying protein structures.

### Comparison of molecular property distributions in LOBSTER to orally bioavailable drugs

**Fig. 1:**
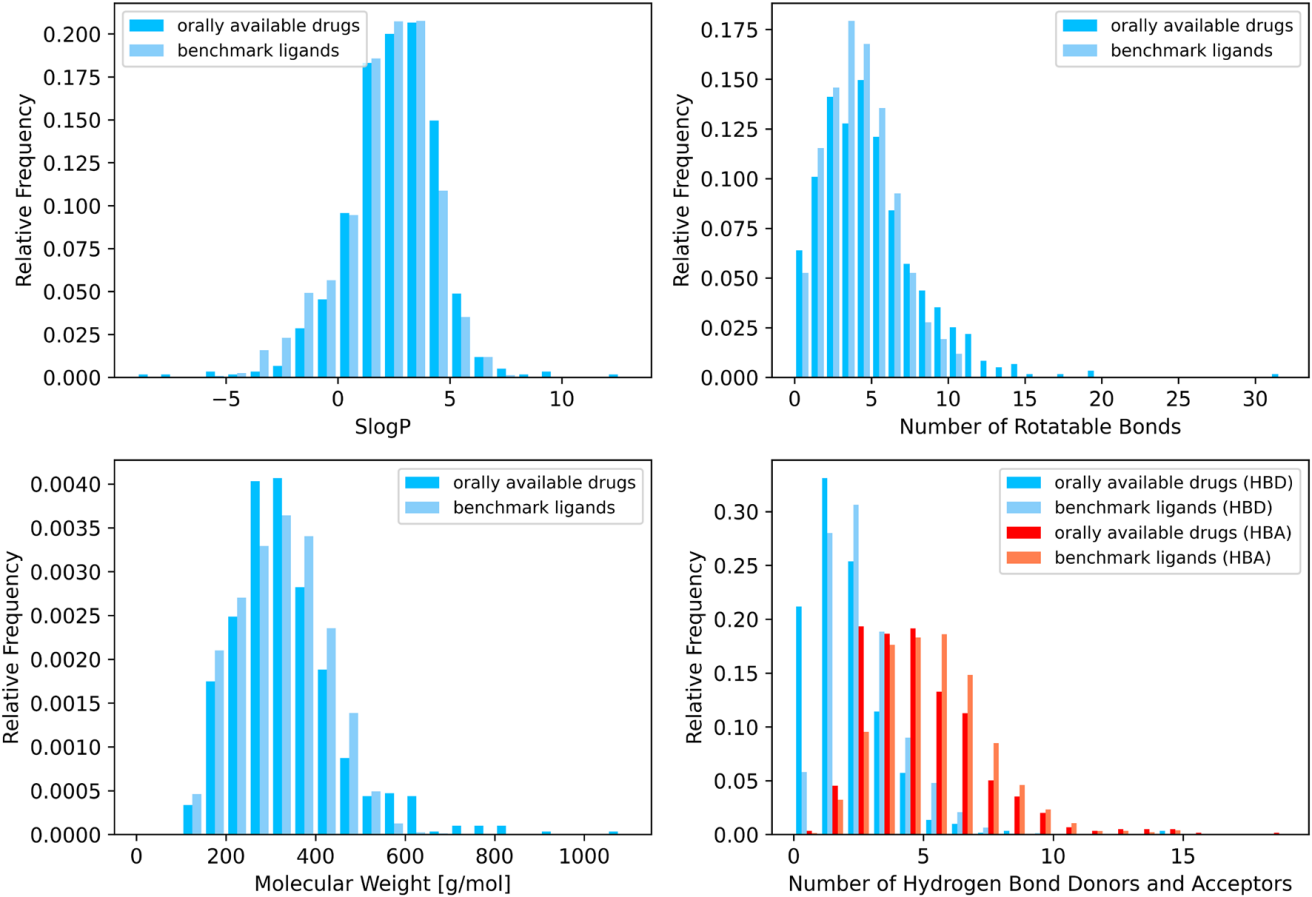
Molecular property distributions of 3212 unique ligands from all ensembles of the LOBSTER dataset and orally available approved drugs as extracted based on the FDA Orange Book and structures of approved drugs from DrugBank. Hydrogen bond donors (HBD) and acceptors (HBA) are shown in blue and red in the last plot, respectively

To investigate the compounds contained in LOBSTER, the physicochemical properties SlogP (predicted), molecular weight, and the number of hydrogen bond donors (HBD) and acceptors (HBA) are analyzed. The number of rotatable bonds represents the molecular flexibility. Figure 1 shows the distributions of these molecular properties for the 3212 unique ligands in a direct comparison to drugs listed in the FDA Orange Book [64].

Overall, LOBSTER covers a reasonably diverse compound collection with distributions of molecular properties reflecting the corresponding ones of the orally availabe drugs. Its size and composition make LOBSTER suitable for statistically meaningful benchmarking. We want to emphasize the relevance of statistically sound benchmarking for improving drug discovery.

### Impact of clustering on the ensemble set

The ensemble dataset is primarily designed to benchmark tools capable of superimposing multiple ligands. Each ensemble is a test case for existing approaches but could also be used to empirically choose the parameters of new scoring functions to assess the quality of molecular overlays. The entire dataset contains 3583 ligands that are incorporated in the analysis below. Of these ligands, 3212 are unique regarding their SMILES representation. To obtain non-intersecting ensembles, we grouped ensembles containing a ligand derived from the same protein-ligand complex into clusters. Here, we evaluate how the clustering affected the LOBSTER set. The distributions of the picked representative ensembles and all clusters are shown with additional figures in the Supplementary Information (SI). The average and median number of molecules in an ensemble is five and three, respectively. Ensembles contain between two and 144 molecules (ensemble name 5L2_A_302-4zx0). The largest ensemble was derived from the pocket of the human carbonic anhydrase II (PDB ID 4ZX0) which is of great pharmaceutical interest [75]. The size of the ensembles varies according to how many pockets with an identical binding site sequence were among the protein-ligand complexes in which a compound from our ligand selection is bound. The larger the ensemble, the more ligands from similar pockets are part of the LOBSTER dataset. The average and median standard deviation in heavy atom count (HAC) among the ligands within an ensemble are 3.18 and 2.74, respectively. Thus, the molecules within the ensembles have comparable sizes. Standard deviations vary from zero to 17.68 (ensemble 0NJ_A_601-4e5d). The two ligands from the ensemble with the highest standard deviation have HACs of 18 and 43 and were extracted from the complexes with PDB IDs 4E5D and 3IES, respectively. In both complexes, the target protein is the firefly luciferase (luciferin 4-monooxygenase, FLuc). FLuc is a reporter frequently used in high-throughput screening assays [76]. Inhibitory compounds are not required to be of pharmacological relevance or to have drug-like physicochemical properties. Only a strong inhibitory activity is of interest. Thus, these compounds can vary in size, explaining the large difference in HAC. The computed clusters had sizes varying from two to 167 ensembles, showing that some binding sites were overrepresented by ensembles before the clustering. These findings confirm that the clustering routine can effectively group ensembles to remove bias from overrepresentation.

For 96 of the 671 clusters, multiple different UniProt accession numbers are present within the cluster. However, multiple Pfam IDs are present for only 11 of these clusters, highlighting that most differences in UniProt accession numbers within these clusters are not due to entirely different proteins in the same cluster but similar ones with identical residues in the binding site. Identical Pfam IDs with multiple UniProt accession numbers can be due to differing organisms (e.g., Q3JY79 and Q9SP37 for cluster RAB_A_60-3glq), virus strains (e.g., C6KP13 and C3W5S3 for cluster LNV_B_801-3ti3 or proteins with identical binding sites (e.g., P45984, P53779 and P45983 for cluster 880_A_502-4z91). The reasons for all clusters with multiple Pfam IDs are explained in Table 2 of the SI. The AZ dataset was analyzed likewise to compare the outcome of our clustering procedure to the protein grouping performed by Giangreco et al. [24]. In the AZ dataset, all clusters contain only a single UniProt accession number by design.

Overall, the SIENA search delivers ensembles of manageable size that each include molecules with a comparable number of heavy atoms. The analysis of the clusters shows that the clustering procedure effectively groups ensembles generated from a similar composition of proteins. Thus, it reduces redundancy without losing valuable data and significantly contributes to our goal of offering diverse data with LOBSTER.

### Analysis of dataset diversity

**Fig. 2:**
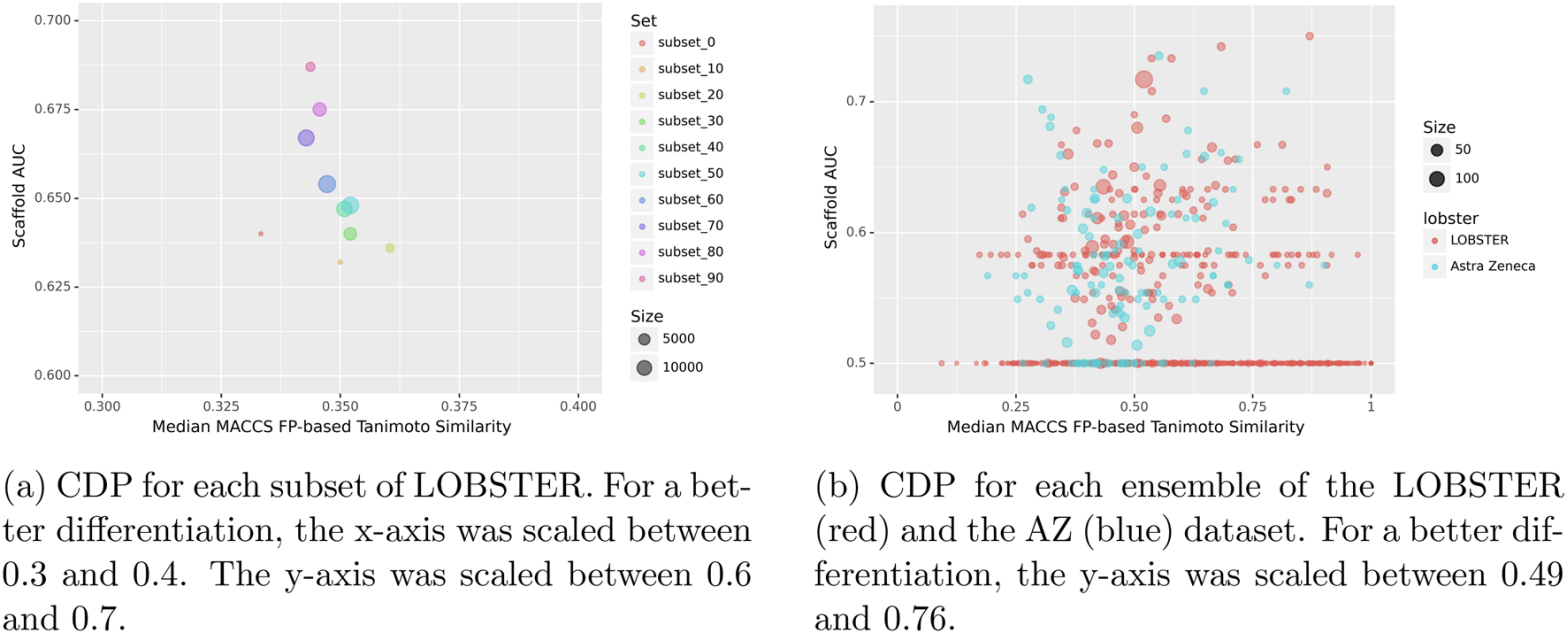
Consensus Diversity Plot (CDP) for **a** each subset of LOBSTER, e.g., *subset_80* includes all pairs having an overlap percentage $80 \le x < 90$, **b** for each ensemble of LOBSTER and the AZ dataset. Six molecules of the AZ dataset in SDF format could not be processed by RDKit and were excluded from the analysis. Dots were scaled in the size of the subset or ensemble of the respective dataset

To evaluate the ratio of Bemis–Murcko scaffold diversity (x-axis) and median MACCS fingerprint similarity (y-axis) of the ligands, a Consensus Diversity Plot (CDP) as described by González-Medina et al. [68] gives an impression of the compound diversity within the dataset. In the CDP in Fig. 2a, all subsets are in the left quadrant of the plot. A higher Scaffold AUC of the Bemis–Murcko Scaffolds indicates less diverse substructures, so values closer to 0.5 on the y-axis represent subsets with more unique scaffolds. A trend of increasing Scaffold AUC with decreasing overlap is visible. The analysis also reveals that all subsets have a low median 2D MACCS fingerprint similarity. However, there is no clear correlation between the 2D similarity and the overlap of the pairs. Figure 2b depicts the CDP for all ensembles of the LOBSTER and AZ datasets to compare the diversity of the ligands within their ensembles. The ensembles show different compositions of ligands concerning the MACCS fingerprint similarity with a Scaffold AUC of at most 0.75. For 464 ensembles, the Scaffold AUC was 0.5. Comparing these findings to the CDP for the AZ dataset, the spread for the median MACCS fingerprint along the x-axis is comparable to the LOBSTER set. Within the AZ set, 25 ensembles have a Scaffold AUC of 0.5. Distinct plots for the ensembles of both datasets are shown in the SI. The overall scaffold diversity of all molecules from the data set is 0.70 for LOBSTER and 0.64 for AZ and, therefore, slightly better in the AZ dataset. The overall median MACCS fingerprint similarity is 0.34 in the LOBSTER and 0.35 in the AZ dataset.

**Fig. 3:**
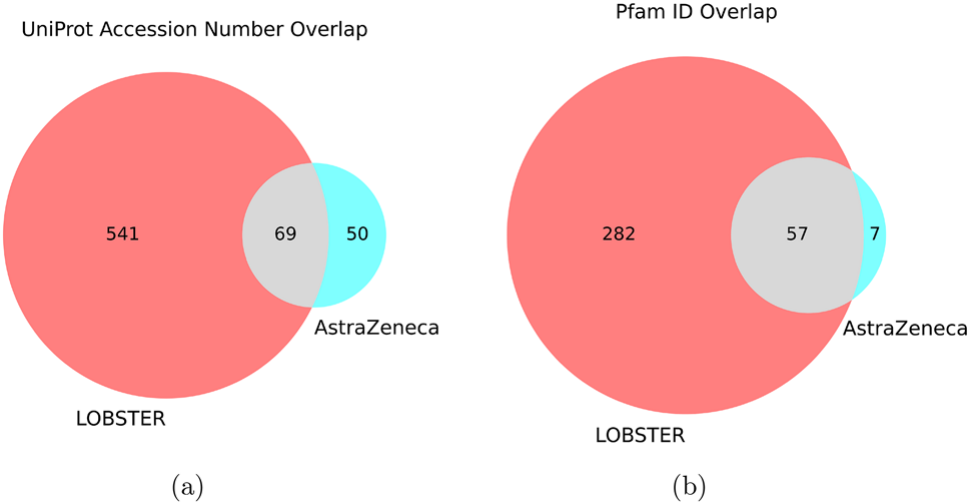
Venn diagrams of the overlap between the LOBSTER dataset and the AZ dataset in terms of **a** UniProt accession numbers and **b** Pfam IDs

The protein families were annotated and compared for both the LOBSTER and the AZ datasets to assess the diversity of the LOBSTER dataset in terms of not only the ligands but also their binding partners. The LOBSTER dataset not only comprises more clusters but also more targets (Fig. 3a). The diversity of protein families can be derived from Fig. 3b. The LOBSTER dataset almost completely encompasses all UniProt accession numbers and Pfam IDs that are part of the AZ dataset while vastly expanding the protein space.

**Fig. 4:**
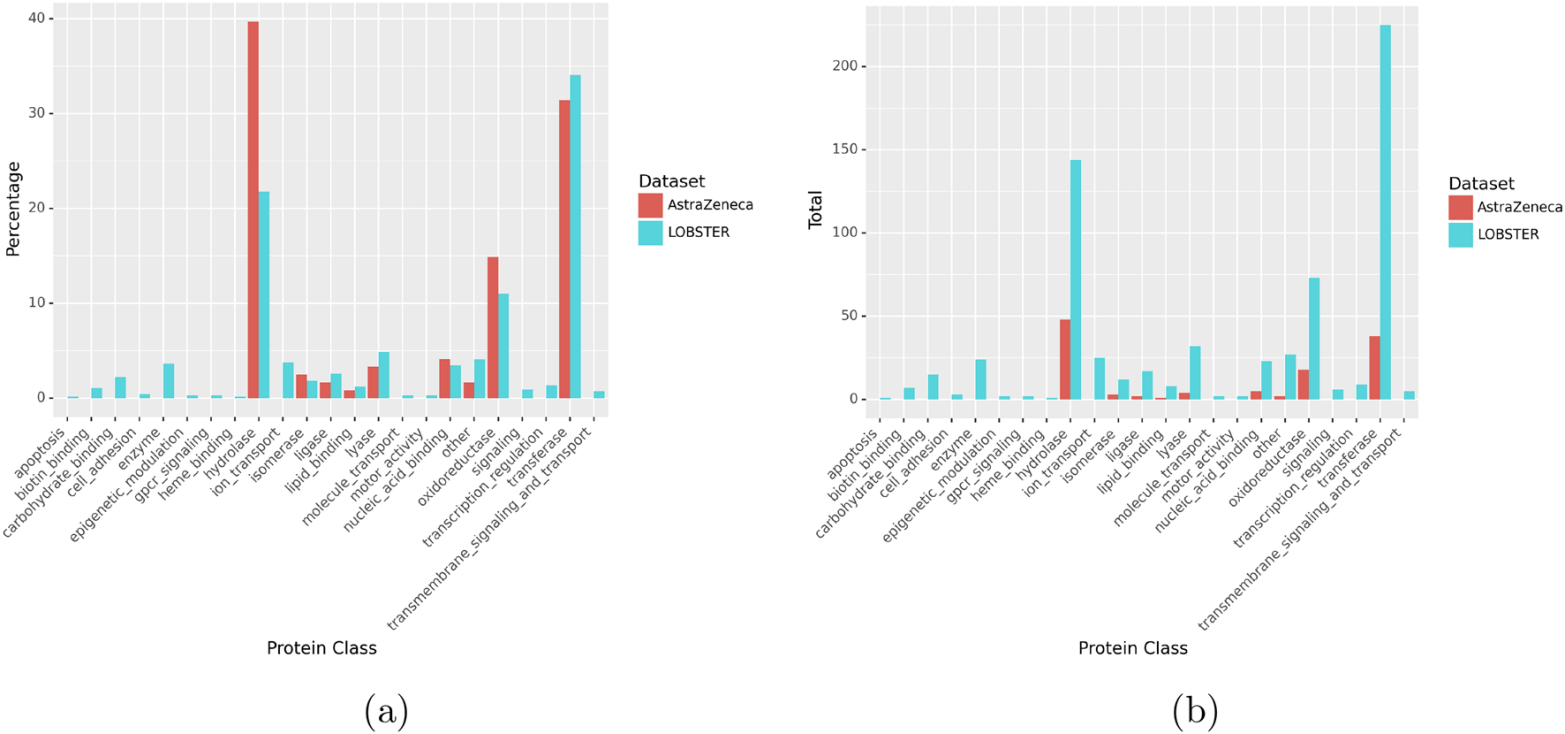
Column diagrams of manually assigned protein classes based on the EC numbers and GO annotations of Pfam IDs. Column diagrams of **a** total numbers of clusters and **b** percentage of clusters corresponding to a given protein class are shown

Using manually assigned protein classes based on EC numbers and GO annotations (a complete list is available in the SI), it becomes apparent how much diversity is gained in the LOBSTER dataset due to the automated processing of the entire PDB. As can be seen in Fig. 4, LOBSTER comprises many new protein classes. But even when neglecting size and focusing on the diversity and, therefore, the frequency of given protein classes, the AZ dataset only shows higher percentages of clusters for the hydrolase, isomerase, and oxidoreductase classes. While this is only a rough categorization, the vast gain in size and diversity are compelling arguments for the LOBSTER dataset.

Overall, LOBSTER covers a wide range of proteins, many of them with relevance in drug discovery. The automated filtering of the entire PDB data strongly contributes to this success. It also leads to a remarkable size and ligand diversity with respect to both the novelty in terms of scaffolds and the features in terms of MACCS fingerprints. While the ligand diversity is comparable to the state-of-the-art AZ dataset, the protein diversity vastly exceeds it.

### Data distribution within the subsets

**Fig. 5:**
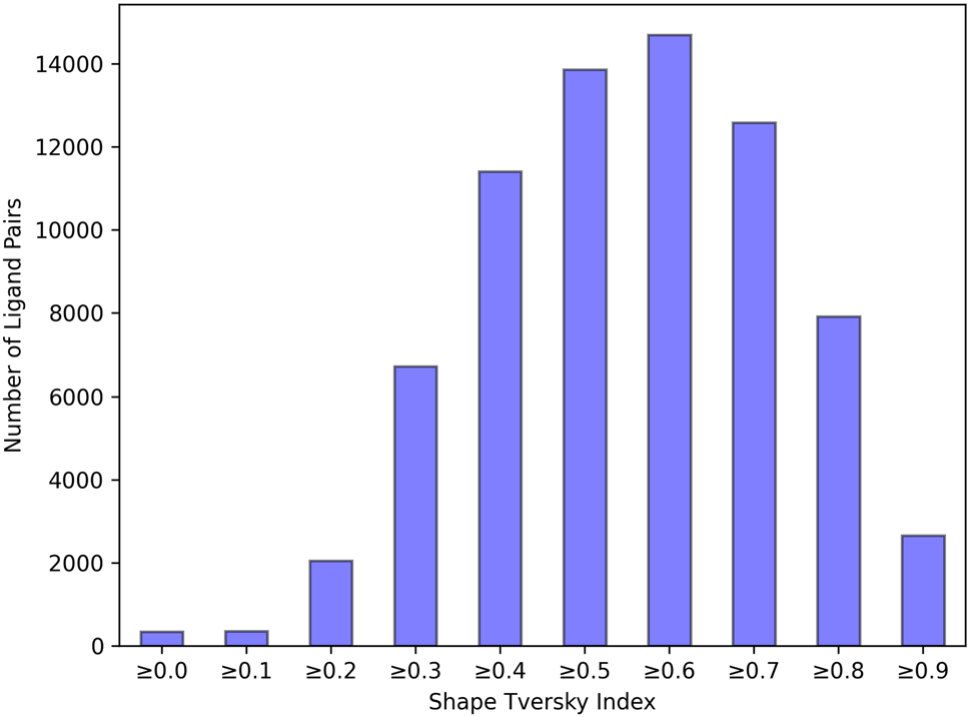
Comparison of the size of the subsets. Labels at the x ticks correspond to the respective Shape Tversky Index bin of the subset, i.e. $\ge 0.8$ corresponds to the set *subset_80*

Figure 5 shows the sizes of the subsets. Most pairs have a Shape Tversky Index of approx. 0.6 and therefore an estimated overlap of 60 % (*subset_60*).

**Fig. 6:**
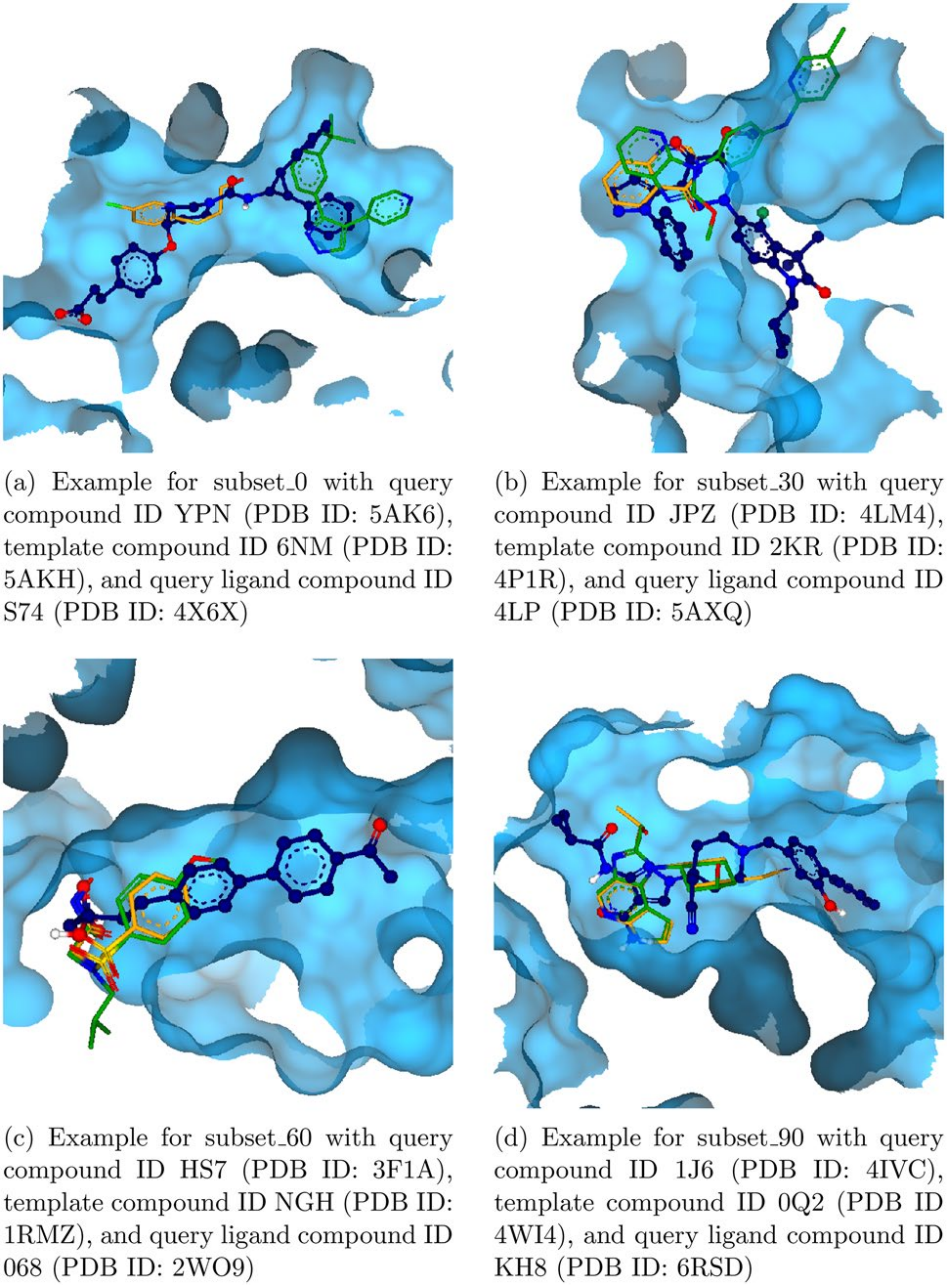
Examples of the subsets for volume overlaps 0, 30, 60 and 90. The query compound is shown in orange and the template in green. The query ligand for the respective SIENA search is shown in dark blue with the surface of its binding pocket in light blue

For the subsets 0, 30, 60, and 90, the overlay of one pair each is shown in Fig. 6. The set *subset_0* is considered extremely challenging because the pairs have a shape overlap of less than 0.1. The occurrence of such pairs can be explained when analyzing the pair in combination with the search ligand used to generate the respective ensemble via SIENA. When visualizing pairs of the *subset_0* with the search ligand, it is visible that the ligands of these pairs describe different parts of the pocket which are connected by the pocket of the search ligand. Figure 6a shows an example of such a ligand superposition. Thus, this subset represents counterexamples that cannot be superimposed by alignment tools. At the same time, the *subset_90* corresponds to an overlap of 0.9 or more and, therefore, gives a good starting point for empirical studies of good superpositions. Overall, the current dataset of all subsets comprises 72,734 ligand pairs. Of these pairs, 66,890 pairs consist of ligands with rotatable bonds and may be of interest for benchmarking fully flexible alignment methods.

### Data distribution over time

**Fig. 7:**
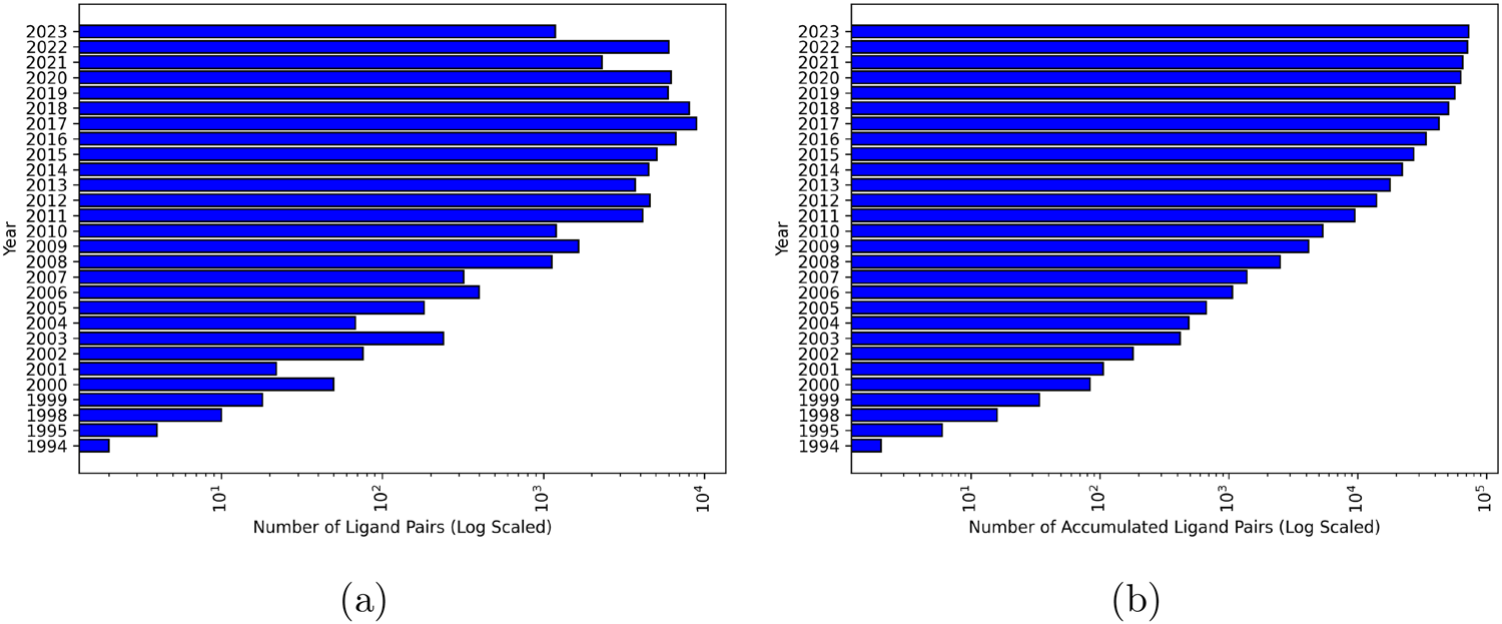
Data distribution of the available ligand pairs generated from the representative ensembles in the LOBSTER set over time. The x-axis is scaled logarithmically to visualize the smaller values. **a** All pairs that became available in the respective year were counted. **b** The number of available pairs within the respective year is shown

Analyzing the hypothetical growth of the LOBSTER dataset over time highlights the advantages of an automated and therefore updateable dataset. It also offers an easily adaptable basis for generating potential time split analyses. Figure 7a shows the 72,734 ligand pairs according to their latest date of the first revision from the underlying two protein files (release date). Additionally, Fig. 7b shows how the ligand pairs accumulate and how the LOBSTER dataset grows over time. Further statistics on the release date and the distribution of molecular properties used for filtering can be found in the SI.

The earliest release date of pairs in LOBSTER is 1994. There are no pairs in 1996 and 1997. No ligand from a PDB structure released in 1996 is part of LOBSTER. A ligand from a PDB structure released in 1997 (PDB ID 1AQ1) is part of the dataset but was counted in pairs with a newer latest release date. The oldest PDB structure used for LOBSTER is from 1993 (PDB ID 1GCA). Most pairs became available in 2017 (8910 pairs), and the smallest number of pairs was counted for 1994 (2 pairs). These may originate from fragment screens. The statistics show how the LOBSTER dataset steadily gains data over time. This continuous growth adds value to the options of versioning and updating available datasets. Both are possible with the resources provided along with this manuscript.

## Conclusion

The LOBSTER dataset was generated by a fully automated protocol to facilitate the development of novel approaches and the benchmarking of existing methods for small molecule superposition. The final LOBSTER set consists of 671 ligand ensembles and 3212 unique ligands from which 72,734 ligand pairs can be derived. Due to its size and diversity, the LOBSTER dataset allows for empirical studies. Automatization allows for objectively selecting the largest set of ligands from the PDB and refining this selection by filtering for drug-like molecules with high ligand efficiency according to activities and affinities from PDBbind [54, 55] and BindingDB [56, 57]. Looking beyond the horizon of the Rule of Five, our filter criteria were designed according to recent literature. Thus, they give a modern twist to the compound selection and make LOBSTER an appropriate and timely choice for systematic evaluations. To offer a basis for objective metrics on superposition experiments and to ensure that the resulting ligand overlays are biologically meaningful, the ligands were superimposed in their crystal pose in so-called ensembles through an alignment of the respective binding pockets. We group ensembles sharing at least one protein-ligand complex into clusters and consider only one representative ensemble per cluster. Thereby, redundant ensembles are eliminated from the dataset, and the diversity of the overlays is increased.

We shared our insights into the data by analyzing the molecular properties, ensemble properties, and cluster composition. The difficulty in reproducing or scoring the biologically meaningful ligand alignments varies tremendously since a broad scope of proteins serves as the basis for ensemble creation. The similarity of the molecules within the ensembles differs significantly, ranging from almost identical to entirely dissimilar. The latter is challenging because predicting the correct overlay might be ambitious for methods relying on a high 2D similarity. To offer further applications besides the ensemble overlays, all pairs of molecules from each ensemble were grouped into ten subsets by their volume overlap, assessed by the Shape Tversky Index. The subset indicates how challenging the corresponding overlays may be to reproduce for superposition methods. An overview of the growth of LOBSTER over time is presented to give further insights into the composition of the dataset. Finally, an analysis of the compound and protein diversity reveals that the LOBSTER set is large and diverse, not only in its selection of ligands but also in the range of protein targets of the respective complexes.

Through the open-source Python scripts, the generation of the LOBSTER set is reproducible and allows for updates, e.g., in the form of an adjustment of the source data. The versioned deployment of the LOBSTER set makes it easy to keep track of changes. Offering recent, refined, and publicly accessible high-quality data in the field of small molecule superposition, the LOBSTER set and its many possible applications will hopefully serve as a basis for comparative benchmarking and further method development.

## Supplementary Information

Below is the link to the electronic supplementary material.

Supplementary file 1 (pdf 2634 KB)

## Data Availability

The LOBSTER dataset is available at https://doi.org/10.5281/zenodo.12658320, including the data tables resulting from LigandFinder and StructureProfiler. All scripts required to generate LOBSTER on the basis of the NAOMI library and RD Kit are available from the GitLab repository at https://github.com/rareylab/LOBSTER.
