# Supplementary material for: Combining crystallographic and binding affinity data towards a novel dataset of small molecule overlays
Source: J Comput Aided Mol Des. 2024 Dec 4;39(1):2. doi: 10.1007/s10822-024-00581-1 (PMC11618164; doi:10.1007/s10822-024-00581-1)
Supplement: Supplementary file 1 — (pdf 2634 KB) [file 10822_2024_581_MOESM1_ESM.pdf]

Supplementary Information: Combining Crystallographic and Binding Affinity Data Towards a Novel Dataset of Small Molecule Overlays

Sophia M. N. Hönig orc,†,‡ Torben Gutermuth orc,‡ Christiane Ehrt orc,‡ Christian Lemmen orc,† and Matthias Rarey orc*,‡

†*BioSolveIT, An der Ziegelei 79, 53757 Sankt Augustin, Germany*

‡ *University of Hamburg, ZBH - Center for Bioinformatics, Albert-Einstein-Ring 8-10, 22761 Hamburg, Germany*

E-mail: matthias.rarey@uni-hamburg.de

Ligands Skipped by Construction

Most ligands were removed during calculations with the NAOMI Framework[1,2] as these ligands were not of interest. Among others, metal ions and covalent ligands were out of scope for this study and were therefore skipped by construction (> 1.5 mio. sites). The following reasons lead to a rejection of ligands:

- ligand is a metal ion
- ligand is not listed in the HET section
- ligand contains a metal ion (e.g. HEM)

- ligand has missing atoms (as listed in REMARK470/REMARK610)
- ligand is split into multiple parts upon reading by NAOMI
- ligand has a HET code that is not listed in the "component.cif" file which is part of the PDB file mirror
- ligand has a molecular formula that is different from the "component.cif"
- ligand is a polymer but monomers come from different chains
- ligand is a polymer but seems to be part of broken larger chain
- ligand is a polymer with missing coordinates
- ligand misses connections recorded in the LINK section
- ligand is part of the protein
- ligand was not built and NAOMI skipped at least one atom by AltLoc
- ligand is listed in the HET section but absent
- ligand represents an unknown atom or ion (three-letter residue name UNX)
- ligand is not present in MODEL 1 that is used by NAOMI

SIENA Settings

For generating binding site ensembles, SIENA 1.3.0[3,4] was applied with the following settings:

- radius 6.5
- identity 1
- bb_rmsd 1
- fragment_length 10
- fragment_distance 4
- resolution 2.5
- flexibility_sensitivity 0.6
- holo_only
- filter_unwanted_ligands false

Remark on the Reliability of Metadata from BioAssays

An analysis of the assay data from PubChem BioAssay[5] used to compile the LIT-PCBA dataset[6] reveals that several assays do not assess the activity on a single protein target, but on numerous pathway-related potential targets whose inhibition might lead to the measured readout in the bioassay. Our findings for the assays included in the LIT-PCBA dataset are summarized below in Table 1.

Table 1: Exemplary assay data from PubChem BioAssay included in the LIT-PCBA dataset.

PubChem BioAssay AID	Annotated PubChem BioAssay Target	PubChem BioAssay Assay Annotation from BioAssay Research Database	Assay Annotation Based on Assay Description	Conclusion
492947	beta-2 adrenergic receptor	cell-based format, protein-protein interaction assay		single target, but cell-based
1030	aldehyde dehydrogenase 1 family, member A1	biochemical format, direct enzyme activity assay		single target
743075	estrogen nuclear receptor alpha	n/a	pathway modification	multiple potential targets
743080	estrogen nuclear receptor alpha	n/a	pathway modification	multiple potential targets
588795	flap endonuclease 1	single protein format, direct enzyme activity assay		single target
2101	glucocerebrosidase	tissue-based format, direct enzyme activity assay		single target, but tissue-based

Continued on next page

Table 1 – continued from previous page

PubChem BioAssay AID	Annotated PubChem BioAssay Target	PubChem BioAssay Assay Annotation from BioAssay Research Database	Assay Annotation Based on Assay Description	Conclusion
602179	isocitrate dehydrogenase 1, partial	biochemical format, coupled enzyme activity assay	mutant	single target
504327	histone acetyltransferase KAT2A isoform 1	protein format, protein-protein interaction assay		single target
995	mitogen-activated protein kinase 1	cell-based format, second messenger assay	pathway modification	multiple potential targets
493208	serine/threonine-protein kinase mTOR isoform 1	cell-based format, phosphorylation assay	pathway modification	multiple potential targets
1777	kappa-type opioid receptor isoform 1	cell-based format, functional assay		single target, but cell-based
1631	pyruvate kinase PKM isoform a	single protein format, direct enzyme activity assay		single target

Continued on next page

Table 1 – continued from previous page

PubChem BioAssay AID	Annotated PubChem BioAssay Target	PubChem BioAssay Assay Annotation from BioAssay Research Database	Assay Annotation Based on Assay Description	Conclusion
743094	peroxisome proliferator activated receptor gamma	n/a	pathway modification	multiple potential targets
651631	Cellular tumor antigen p53	n/a	pathway modification	multiple potential targets
504847	vitamin D3 receptor isoform VDRA	biochemical format, protein-protein interaction assay		single target

Assignment of Protein Classes Based on PFam IDs

The assignment of protein classes based on the corresponding Pfam IDs was done using a KNIME[7] workflow. We used the SIFTS[8]-derived associations between PDB chains and EC numbers and gene ontology (GO)[9] identifiers to assign classes to the respective Pfam IDs. First, we assigned all Pfam IDs of chains with an enzyme classification (EC) number as enzymes and categorized them based on the six reaction types they catalyze. If we found multiple reaction types per protein chain, the chain was classified as an enzyme. For the remaining chains, we used a hierarchical classification into ion transport

proteins (GO:0015276, GO:0006811, GO:0005216, GO:0006826, GO:0035725, GO:1902358), G-protein signaling proteins (GO:0007186, GO:0007186), proteins involved in transcription regulation (GO:0006357, GO:0006412, GO:0000122, GO:0006355, GO:0004879), nucleic acid-binding proteins (GO:0003723, GO:0003729, GO:0003677, GO:0004812, GO:0002161), carbohydrate-binding proteins (GO:0030246, GO:0005975), lipid-binding proteins (GO:0008289), biotin-binding proteins (GO:0009374), proteins with motor activity (GO:0003774, GO:0003777, GO:0000146), transmembrane transport and signaling proteins (GO:0004888, GO:0055085, GO:0015234), proteins involved in cell adhesion (GO:0007155), proteins involved in signaling (GO:0007264, GO:0005085, GO:0003924, GO:0009738, GO:0009736), epigenetic modulators (GO:0006338, GO:0016586), heme-binding proteins (GO:0020037), proteins responsible for molecule transport (GO:0015888, GO:0015846), and apoptosis-related proteins (GO:0042981). For some Pfam IDs, we found several assigned classes. Then, we used the most often occurring class for the corresponding protein chains to obtain unique associations between Pfam IDs and our assigned protein classes. For identical percentages, we preferred catalyzed reactions over enzyme over GO-based annotations.

Illustration of Ensemble and Cluster Properties

To evaluate how the clustering affected the LOBSTER set, Figure 1 shows distributions of the picked representative ensembles as well as all clusters as an addition to the main text.

To assess the diversity of the LOBSTER ligands independent of their 3D conformations, the average Tanimoto similarity of the molecules was calculated using the Morgan fingerprint[10] with radius two, which corresponds to the ECFP4. Additionally, the average Tanimoto similarity with the Gobbi 2D pharmacophore fingerprint[11] was used to assess pharmacophore-based similarity. Both measures were used as implemented in RDKit version 2022.09.1.[12] For each ensemble, all molecules were compared once to all other molecules in the ensemble and once to the respective search ligand of the ensemble, since the pocket

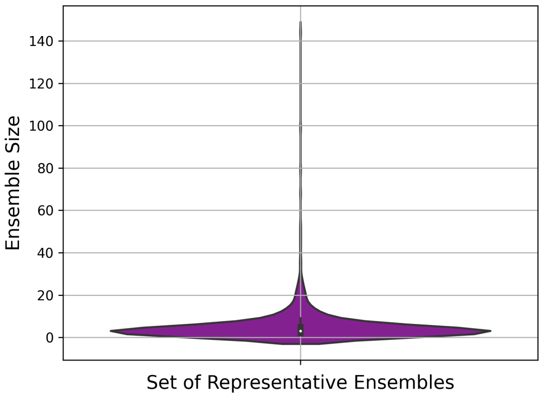

(a)

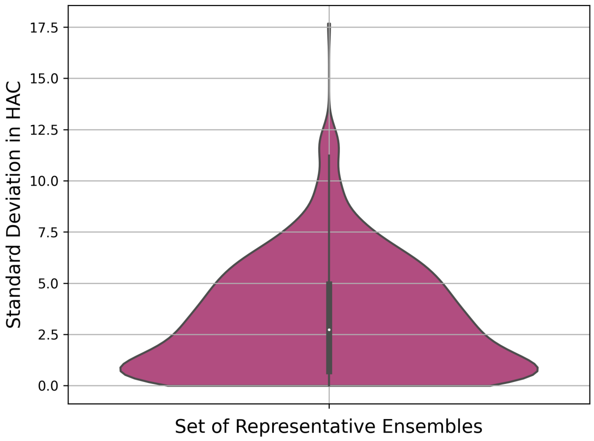

(b)

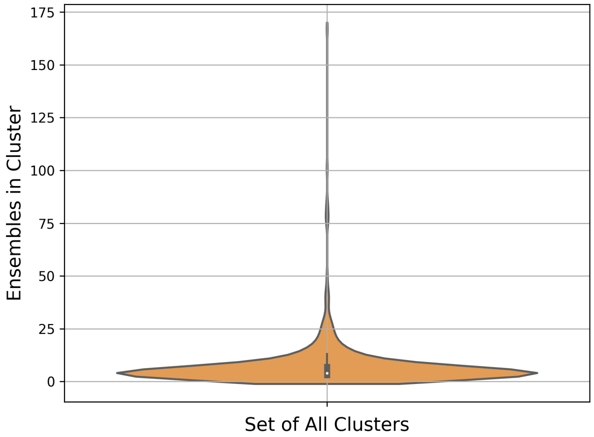

(c)

Figure 1: Distributions of (a) the number of ligands per representative ensemble, (b) the standard deviation of the heavy atom count (HAC) of the ligands per representative ensemble, and (c) the number of ensembles per cluster.

of this ligand is connecting the non-intersecting subpockets of all other ligands within the ensemble. The results using both fingerprints and both comparison strategies are shown in Figure 2. The Tanimoto coefficients based on both fingerprints indicate a low 2D similarity of the molecules with an average and median value below 0.3.

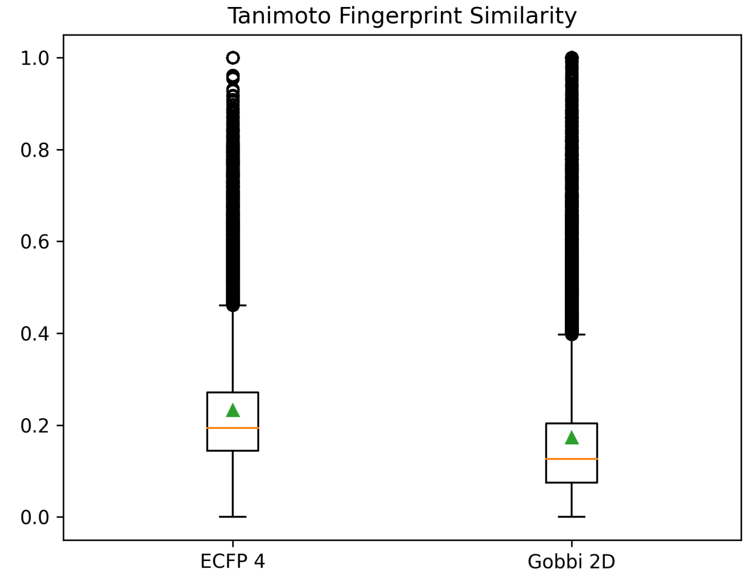

Figure 2: Tanimoto fingerprint similarity for ECFP4 and Gobbi 2D pharmacophore fingerprint (Gobbi 2D). For each ensemble, all molecules were compared to all other molecules in the ensemble (Ensemble). Additionally, all molecules of each ensemble were compared to the respective ensemble's search ligand (Search Ligand). Each data point corresponds to the average value of these comparisons for one ensemble. The orange line marks the median; the green triangle marks the average of all ensembles.

Further Development of the LOBSTER Set Over Time

In the main text, an example time split by the year of the PDB file's first revision (release date) was shown for the set of all ligand pairs within the LOBSTER dataset. Figure 3 shows statistics over all ligand molecules of the LOBSTER dataset, again split by first revision date of the PDB file. The analyzed properties were the quantitative filters applied in our refinement protocol. Please note that the ligand efficiency is not included, since it is

considered a qualitative filter because the normalized values of affinities and activities, which were the basis for the calculation, were derived from different sources and should thus not be compared among the ligands.

Additions to Dataset Diversity Analyses

Some of the data points from the visualization of the LOBSTER and the AZ dataset were overlapping in the Consensus Diversity Plot (CDP) shown in Figure 7 of the main text. Figure 4 shows the ensembles for both datasets in two distinct CDPs to allow a more detailed visualization.

For the analysis of protein diversity and a comparison to the AZ dataset, UniProt accession numbers and Pfam IDs were analyzed in the main text. Figure 5 additionally shows the overlap of the Pfam names from the LOBSTER and AZ dataset.

Cluster analysis

As an extension of the cluster analysis in the main text, Table 2 elucidates clusters within the LOBSTER dataset, which consist of ligands interacting with protein chains with multiple Pfam IDs.

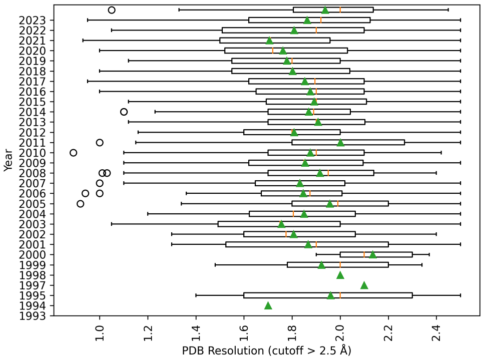

(a)

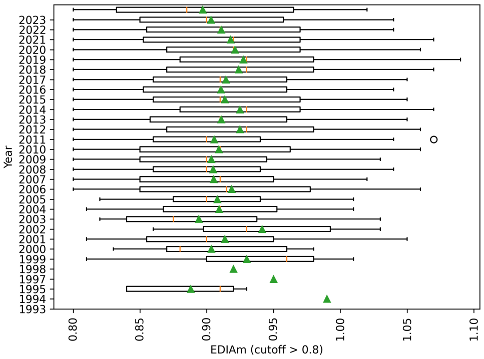

(b)

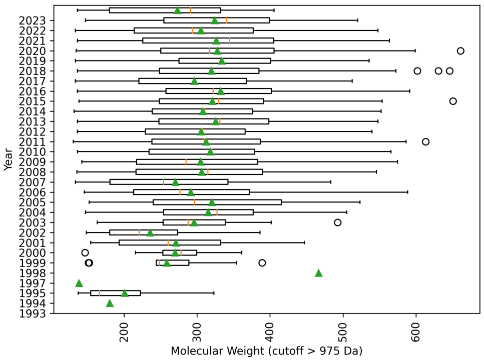

(c)

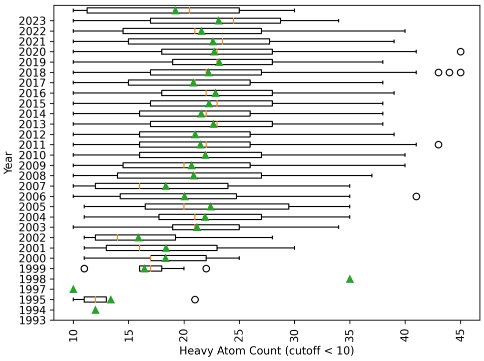

(d)

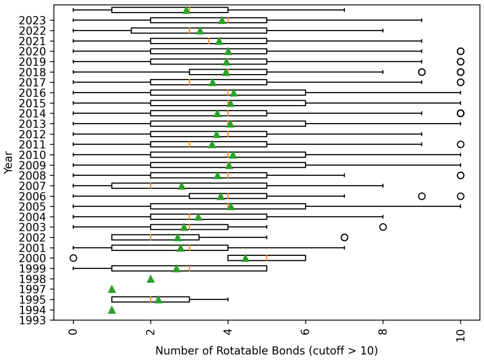

(e)

Figure 3: Time split of the molecules in the LOBSTER dataset according to a) PDB entry resolution, b) $\mathrm{EDIA_m}$ value, c) molecular weight in Da, d) heavy atom count, and e) number of rotatable bonds.

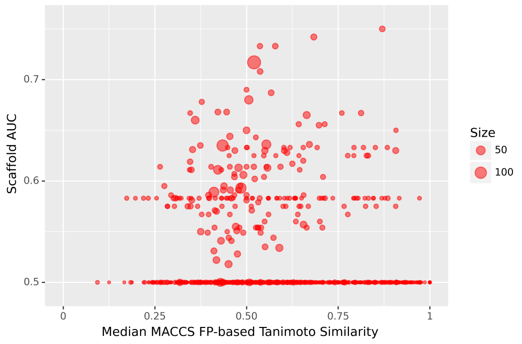

(a) CDP for each ensemble of LOBSTER.

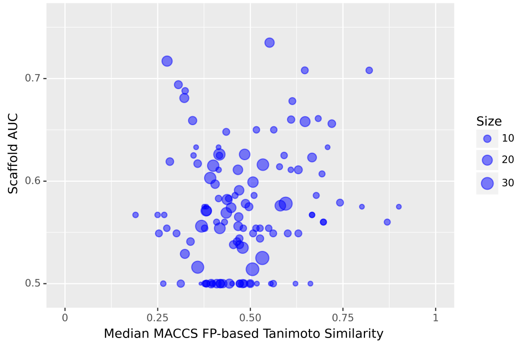

(b) CDP for each ensemble of the AZ dataset.

Figure 4: CDPs for LOBSTER (a) and AZ dataset (b). For a better separation, the y-axis was scaled between 0.49 and 0.76.

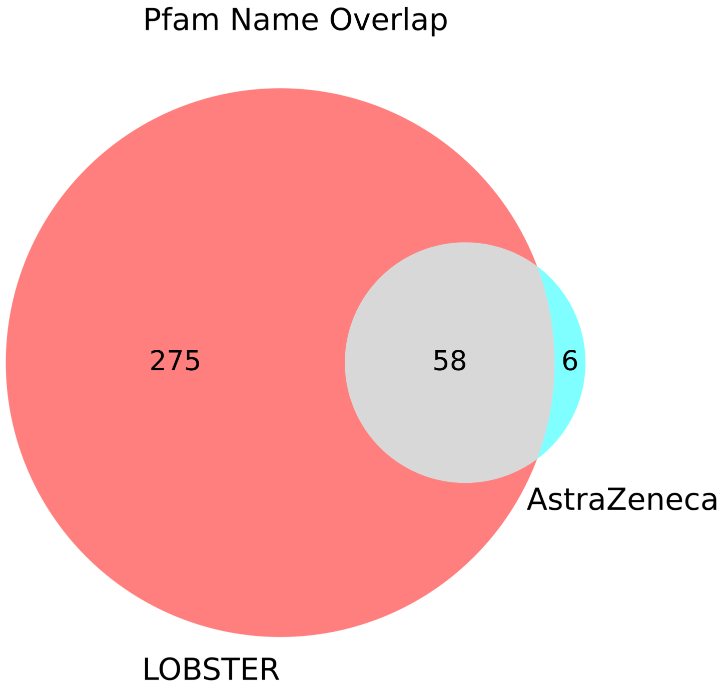

Figure 5: Venn diagram of the overlap between the LOBSTER dataset and the AZ dataset in terms Pfam names.

Table 2: Analysis of the clusters with multiple Pfam IDs

Cluster	UniProt accession numbers	Pfam IDs	Difference
8Y2_A_601-5ni8	P51449, Q15788	PF08832, PF00105	PDB entries 7KXD and 6W9I contain nuclear receptor coactivator 1 peptide in the interacting chain
9FJ_D_503-5vmp	Q16695, O75164	PF00125, PF13832	Chain C in PDB entry 4V2W is interacting with a histone which causes the extra UniProt accession number
GR7_B_201-5wlo	P03369, Q72498	PF00098, PF06817	Protease structures of different strains of human immunodeficiency virus 1
GYN_C_301-2x00	Q8WSF8, P02708	PF02931, PF02932	Different subunits of acetylcholine receptor
HW1_A_800-4cx2	P29476, P29475, P29473, P29474	PF00595, PF02898	NOS1 and NOS3 of different organisms
OAN_D_1627-5a6b	A0A0H2US73, Q6ST21	PF00728, PF18088	Two similar glycosidases
OGN_A_2000-3suu	D0VX21, D2KW09	PF02838, PF13385	Different organisms result in different Pfam IDs
Q51_A_401-6udv	Q07820, P0AEX9	PF01547, PF00452	Protein chimera in PDB entries 4WMU and 4WMX
TIV_B_504-5cb4	P63043, P32882, P81947	PF00836, PF03953	Interacting chain E in 5CB4 matches stathmin family
TSS_A_1433-2iuq	P84888, P84887	PF06433, PF02975	Light and heavy chain of identical target
YC8_B_1-5fp3	O15550, Q5NCY0, O15054	PF21326, PF13181	KDM6A and KDM6B result in different Pfam IDs

References

(1) Urbaczek, S.; Kolodzik, A.; Fischer, J. R.; Lippert, T.; Heuser, S.; Groth, I.; Schulz-Gasch, T.; Rarey, M. NAOMI: on the almost trivial task of reading molecules from different file formats. *Journal of Chemical Information and Modeling* **2011**, *51*, 3199–3207.

(2) Urbaczek, S.; Kolodzik, A.; Groth, I.; Heuser, S.; Rarey, M. Reading PDB: perception of molecules from 3d atomic coordinates. *Journal of Chemical Information and Modeling* **2013**, *53*, 76–87.

(3) Bietz, S.; Rarey, M. SIENA: efficient compilation of selective protein binding site ensembles. *Journal of Chemical Information and Modeling* **2016**, *56*, 248–259.

(4) University of Hamburg SIENA : ZBH: University of Hamburg. https://www.zbh.uni-hamburg.de/forschung/amd/software/siena.html, accessed on February 04, 2022.

(5) Kim, S.; Chen, J.; Cheng, T.; Gindulyte, A.; He, J.; He, S.; Li, Q.; Shoemaker, B. A.; Thiessen, P. A.; Yu, B.; others PubChem 2023 update. *Nucleic Acids Research* **2023**, *51*, D1373–D1380.

(6) Tran-Nguyen, V.-K.; Jacquemard, C.; Rognan, D. LIT-PCBA: An unbiased data set for machine learning and virtual screening. *Journal of Chemical Information and Modeling* **2020**, *60*, 4263–4273.

(7) Berthold, M. R.; Cebron, N.; Dill, F.; Gabriel, T. R.; Kötter, T.; Meinl, T.; Ohl, P.; Sieb, C.; Thiel, K.; Wiswedel, B. KNIME: The Konstanz Information Miner. Data Analysis, Machine Learning and Applications. Berlin, Heidelberg, 2008; pp 319–326.

(8) Dana, J. M.; Gutmanas, A.; Tyagi, N.; Qi, G.; O'Donovan, C.; Martin, M.; Velankar, S. SIFTS: updated Structure Integration with Function, Taxonomy and Sequences re-

source allows 40-fold increase in coverage of structure-based annotations for proteins. *Nucleic Acids Research* **2018**, *47*, D482–D489.

(9) The Gene Ontology Consortium et al. The Gene Ontology knowledgebase in 2023. *Genetics* **2023**, *224*, iyad031.

(10) Rogers, D.; Hahn, M. Extended-connectivity fingerprints. *Journal of Chemical Information and Modeling* **2010**, *50*, 742–754.

(11) Gobbi, A.; Poppinger, D. Genetic optimization of combinatorial libraries. *Biotechnology and Bioengineering* **1998**, *61*, 47–54.

(12) RDKit: Open-source cheminformatics. http://www.rdkit.org, version 2022.09.1, accessed 2020-08-13.
